# Exercise Intensity Modulates Glucose-Stimulated Insulin Secretion when Adjusted for Adipose, Liver and Skeletal Muscle Insulin Resistance

**DOI:** 10.1371/journal.pone.0154063

**Published:** 2016-04-25

**Authors:** Steven K. Malin, Corey A. Rynders, Judy Y. Weltman, Eugene J. Barrett, Arthur Weltman

**Affiliations:** 1 Department of Kinesiology, University of Virginia, Charlottesville, VA, United States of America; 2 Division of Endocrinology and Metabolism, University of Virginia, Charlottesville, VA, United States of America; 3 Exercise Physiology Core Laboratory, University of Virginia, Charlottesville, VA, United States of America; 4 Division of Geriatric Medicine, University of Colorado Anschutz Medical Campus, Aurora, Colorado, United States of America; University of Birmingham, UNITED KINGDOM

## Abstract

Little is known about the effects of exercise intensity on compensatory changes in glucose-stimulated insulin secretion (GSIS) when adjusted for adipose, liver and skeletal muscle insulin resistance (IR). Fifteen participants (8F, Age: 49.9±3.6yr; BMI: 31.0±1.5kg/m^2^; VO_2_peak: 23.2±1.2mg/kg/min) with prediabetes (ADA criteria, 75g OGTT and/or HbA_1c_) underwent a time-course matched Control, and isocaloric (200kcal) exercise at moderate (MIE; at lactate threshold (LT)), and high-intensity (HIE; 75% of difference between LT and VO_2_peak). A 75g OGTT was conducted 1 hour post-exercise/Control, and plasma glucose, insulin, C-peptide and free fatty acids were determined for calculations of skeletal muscle (1/Oral Minimal Model; SM_IR_), hepatic (HOMA_IR_), and adipose (ADIPOSE_IR_) IR. Insulin secretion rates were determined by deconvolution modeling for GSIS, and disposition index (DI; GSIS/IR; DI_SMIR_, DI_HOMAIR_, DI_ADIPOSEIR_) calculations. Compared to Control, exercise lowered SM_IR_ independent of intensity (*P*<0.05), with HIE raising HOMA_IR_ and ADIPOSE_IR_ compared with Control (*P*<0.05). GSIS was not reduced following exercise, but DI_HOMAIR_ and DI_ADIPOSEIR_ were lowered more following HIE compared with Control (*P*<0.05). However, DI_SMIR_ increased in an intensity based manner relative to Control (*P*<0.05), which corresponded with lower post-prandial blood glucose levels. Taken together, pancreatic insulin secretion adjusts in an exercise intensity dependent manner to match the level of insulin resistance in skeletal muscle, liver and adipose tissue. Further work is warranted to understand the mechanism by which exercise influences the cross-talk between tissues that regulate blood glucose in people with prediabetes.

## Introduction

Nearly 470 million worldwide have prediabetes [1], with approximately 86 million individuals in the U.S. population being diagnosed [2,3]. Insulin resistance in skeletal muscle, liver and adipose tissue are considered major etiological factors in the conversion from prediabetes to frank type 2 diabetes [4]. However, many people with insulin resistance maintain normal glycaemia due to compensatory rises in pancreatic insulin secretion. Subsequently, maintaining the capacity of β-cells to secrete adequate amounts of insulin in response to multi-organ insulin resistance is paramount to preventing progression from prediabetes to type 2 diabetes [5].

Exercise training reduces glucose-stimulated responses to carbohydrate consumption [6–10]. However, glucose-stimulated insulin secretion (GSIS) is influenced by the prevailing level of multi-organ insulin sensitivity, such that the product of GSIS and insulin sensitivity (i.e. disposition index) may provide a more accurate view of β-cell function [10]. In fact, pancreatic function is considered a better predictor of future diabetes development than insulin sensitivity alone [11–13]. Thus, identifying the optimal dose at which exercise effects pancreatic function is of pressing clinical need [13–16]. Although recent evidence suggests that high intensity exercise training confers high cardiometabolic benefit (e.g. reduction in abdominal visceral fat, total cholesterol and/or blood pressure), less attention has been directed at understanding the dose of exercise required to optimize insulin sensitivity and β-cell function [16–18]. In fact, no study to date has determined the effect of exercise intensity on β-cell function independent of weight loss or enhanced cardiorespiratory fitness. We recently demonstrated that acute high intensity exercise lowered post-prandial blood glucose more than an isocaloric bout of moderate intensity exercise in men and women with prediabetes, but the role of pancreatic function was not assessed [19]. Therefore, the purpose this study was to test the effect of exercise intensity on β-cell function to determine if this change in insulin secretion would correspond with favorable changes in blood glucose. Given that high intensity exercise in our prior work [19] improved postprandial blood glucose more than moderate intensity exercise, we hypothesized that changes in GSIS adjusted for skeletal muscle, liver and/or adipose insulin resistance may in part explain this improved glycemic control response in men and women.

## Materials and Methods

### Participants

These were the same individuals who were included in our prior study on glucose tolerance [19], but only 15 participants were studied here for further analysis on pancreatic function due to technical difficulty with FFA analysis (n = 3). Participants were recruited via advertisements in the local community. Prediabetes was defined as either a fasting plasma glucose between 100–126 mg/dl, 2 hour glucose between 140–200 mg/dl after a 75g oral glucose tolerance test (OGTT), and/or HbA_1c_ values between 5.7–6.4%. All men and women were non-smoking and sedentary (exercise < 30 min/d, < 3 d/wk) and underwent medical history and physical examination that included a resting and exercise stress test with 12-lead electrocardiogram as done previously [20]. Blood and urine chemistry analysis was also conducted to exclude people with type 2 diabetes, liver disease, cardiac dysfunction, pulmonary abnormalities and renal/liver complications. Pre-menopausal women were studied during the early follicular phase (days 2–8) of the menstrual cycle, and participants were excluded if taking medications considered to impact glucose metabolism [21–23]. All participants provided written signed and verbal informed consent and this study was approved by the University of Virginia Institutional Review Board.

### Body Composition and Aerobic Fitness

Weight was assessed on a digital platform with minimal clothing, and height was recorded with a stadiometer. Body fat and fat-free mass was measured using air displacement plethysmography (BodPod, Cosmed, Concord, CA) corrected for thoracic gas volume [20]. Participants completed a VO_2_peak/lactate threshold cycle ergometer test using open-circuit spirometric techniques (Viasys Vmax Encore, Yorba Linda, CA). An indwelling catheter was inserted in a forearm vein and blood samples were taken at rest and at the end of each exercise stage for blood lactate analysis (YSI Instruments 2700, Yellow Springs, OH). Lactate threshold was determined from the blood lactate-power output relationship and was defined as the highest power output attained prior to the curvilinear increase in blood lactate above baseline.

### Metabolic Control

Participants were instructed to consume ~200 g/d of carbohydrate for 72 hours prior to testing. A 3 d food diary was used to record dietary intake each day, and participants were instructed to repeat this same diet for all testing conditions. Participants were also instructed to refrain from alcohol, caffeine, and vigorous physical activity for at least 72 hour prior to their OGTT.

### Exercise/Control Conditions

Participants reported to the Exercise Physiology Core Laboratory on three separate occasions, after a 10–12 hour overnight fast, and completed randomly assigned control (rest for 1 hour) and 200-kcal bouts of moderate intensity exercise (at lactate threshold) or high intensity exercise (75% of the difference between lactate threshold and VO_2_peak). The time required to expend 200-kcal was calculated from VO_2_peak values [19].

### Insulin Resistance and Pancreatic β-cell Function

One hour following exercise or rest conditions, participants received a 75g OGTT. Blood samples were obtained from an antecubital vein for the determination of substrates. Free fatty acids (FFAs) were determined at 0, 30 and 120 min to provide an assessment of lipid metabolism. Glucose, insulin and C-peptide total area under the curve (AUC) during the OGTT was calculated using the trapezoidal rule from data at 0, 30, 60, 90, and 120 minutes as previously performed by our group and others [16,24,25]. Skeletal muscle insulin resistance was calculated using the inverse of the oral glucose minimal model, which has been validated against the euglycemic clamp technique [26,27]. Hepatic and adipose insulin resistance were estimated by multiplying fasting glucose and FFAs by fasting insulin, respectively. Pre-hepatic insulin secretion rate (ISR) was reconstructed by deconvolution from plasma C-peptide [27]. C-peptide was utilized to characterize insulin secretion to minimize influences of insulin clearance on pancreatic function assessment. Thus, GSIS was calculated as AUC of ISR divided by glucose during the OGTT to provide more accurate depictions of insulin secretion [28,29]. Early (0–30 minutes) and total phase (0–120 minutes) β-cell function, or disposition index, relative to skeletal muscle was calculated as GSIS x 1/oral glucose minimal model. β-cell function relative to hepatic and adipose insulin resistance was also calculated as GSIS x (1/HOMA-IR or 1/Adipose-IR). Hepatic insulin clearance was estimated by dividing AUC of C-peptide by insulin during the OGTT.

### Biochemical Analysis

Plasma glucose was analyzed by a glucose oxidase assay (YSI Instruments 2700, Yellow Springs, OH). Remaining samples were stored at -80°C until later batched-analysis and all samples were run in duplicate to minimize variance within conditions. Insulin and C-peptide concentrations were measured using a chemiluminescent immunometric assay (Diagnostic Products Corporation, Immulite 2000, Los Angeles, CA). Plasma FFAs were determined by a colorimetric assay (Wako Chemicals, Richmond, VA).

### Statistical Analysis

Data were analyzed using the statistical program R (The R Foundation, Vienna, Austria 2013). Skewed data were log transformed for statistical analysis to meet normality requirements. Data were compared across conditions using a repeated measures one way analysis of variance. Pairwise comparisons were used to identify the source of significance in the event of a significant main effect. Because men and women typically differ in body composition and differences in body fat may impact insulin secretion, sex was used as a co-variate to confirm the effect of exercise intensity on pancreatic function. Pearson’s correlation was used to determine associations. Data are reported as mean ± standard error of mean or median (IQR) when data were not normally distributed. Significance was accepted as *P*≤0.05.

## Results

### Participant and Exercise Characteristics

Participants were middle-aged (49.9±3.6 yr), obese (BMI: 31.0±1.5 kg/m^2^ and body fat: 40.6±1.5%) and had poor cardiorespiratory fitness (VO_2_peak: 23.2±1.2 mg/kg/min). Participants also had on average normal fasting glucose and impaired glucose tolerance following the 75g OGTT (2 hour glucose: 170.2±8.8 mg/dl; Table 1***)***. Individuals performed moderate intensity exercise (67.5±1.4% of VO_2_peak for 41.6±2.3 min) and high intensity exercise (90.2±1.3% of VO_2_peak for 23.7±1.3 min) until 200-kcal were expended.

**Table 1:** Subject Characteristics.

	Control	Females	Males
N, (M/F)	15	8F	7M
Age (years)	49.9 ± 3.6	48.1 ± 5.2	52.0 ± 5.4
Height (cm)	169.2 ± 2.9	161.8 ± 2.9	177.7 ± 3.0*
Body weight (kg)	91.6 ± 4.5	85.4 ± 7.3	98.7 ± 3.9
Body mass index (kg/m^2^)	31.0 ± 1.5	32.5 ± 2.5	30.0 ± 1.6
Fat mass (kg)	37.5 ± 2.8	37.4 ± 4.5	37.6 ± 3.5
Fat-free mass (kg)	54.0 ± 2.4	47.9 ± 3.0	61.0 ± 1.7*
Body fat (%)	40.6 ± 1.5	43.1 ± 1.7	37.7 ± 2.4
VO_2_peak (L/min)	2.1 ± 0.1	1.9 ± 0.1	2.3 ± 0.1
VO_2_peak (mg/kg/min)	23.2 ± 1.2	23.1 ± 1.7	23.4 ± 1.9
*OGTT Screening*			
FPG (mg/dl)	105.2 ± 2.9	105.3 ± 3.9	105.0 ± 4.8
2-hour PG (mg/dl)	170.2 ± 8.8	170.2 ± 8.8	155.0 ± 13.9
HbA_1c_ (%)	5.7 ± 0.1	5.7 ± 0.1	5.6 ± 0.1

Data are expressed as mean ± SEM. Sex differences were compared with independent *t*-test.

*Compared with Females (P<0.05).

### Glucose, Insulin and FFA Metabolism

**Fig 1:**
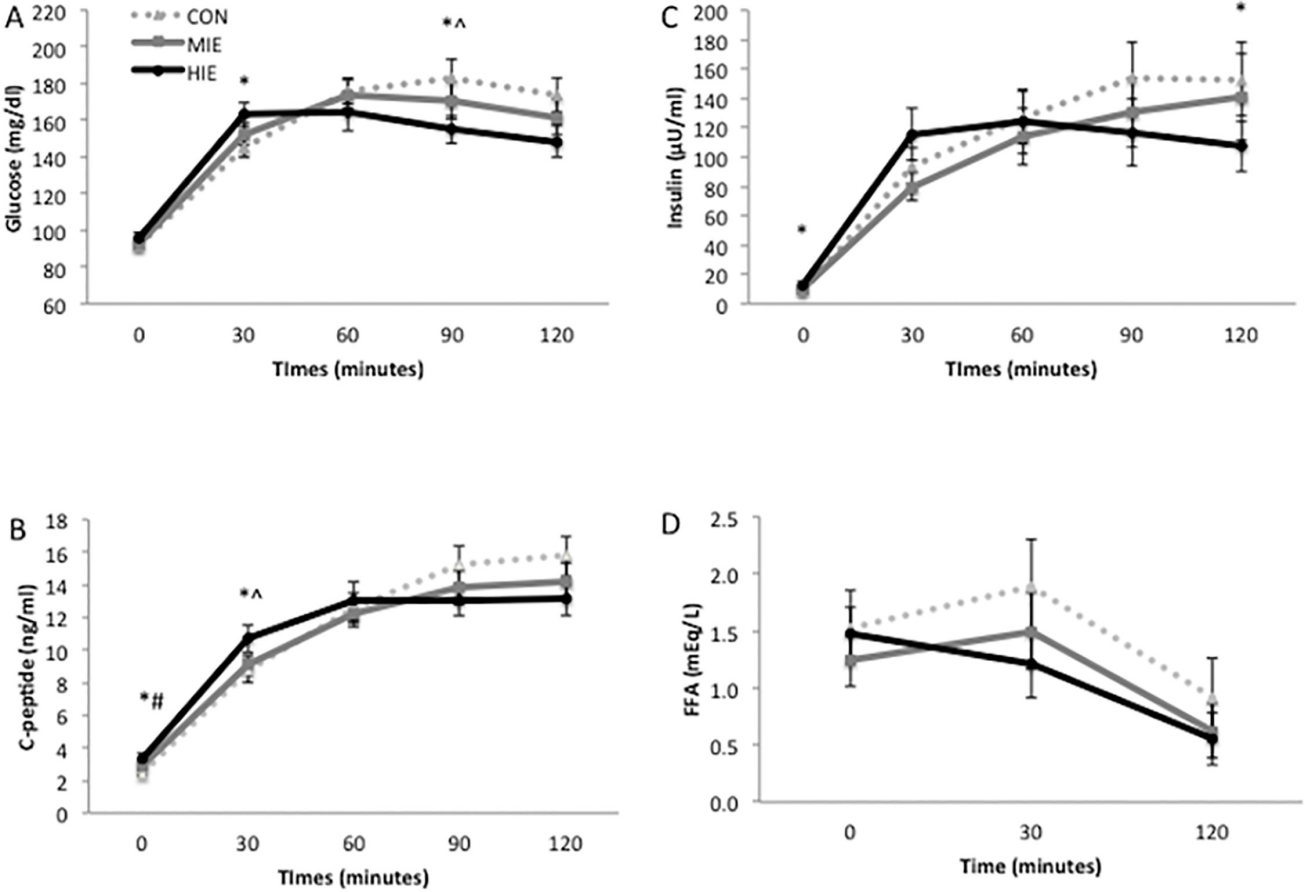
Effect of exercise intensity on plasma glucose, insulin, C-peptide and FFA. FFA = free fatty acids. *Compared to Control, P<0.05. ^Compared to MIE, P<0.05.

As shown in our previous report [19], exercise-induced reductions in post-prandial glucose concentrations following high compared with moderate intensity exercise and Control (Fig 1). Plasma insulin was significantly higher prior to the OGTT following high intensity exercise compared to Control, although insulin levels were lower towards the later portion of the test (Fig 1). Fasting FFA concentrations were not statistically different across conditions, although exercise reduced FFA AUC following moderate and high intensity exercise (P<0.05, Table 2).

**Table 2:** Area under the curve from the OGTT before and after exercise at different intensities.

	Control	MIE	HIE
*Early Phase Responses*			
Glucose AUC_0-30_ (mg/dl*30min)	3561.5 ± 100.0	3672.1 ± 117.1	3882.0 ± 141.9*
Insulin AUC_0-30_ (μU/ml*30min)‡	1358 [1001, 1990]	1388.0 [846.6, 1611]	1538 [1097, 2576]*
C-peptide AUC_0-30_ (ng/ml*30min)	168.2 ± 11.7	180.1 ± 11.7	210.4 ± 13.8*^
ISR AUC_0-30_ (ng/ml*30min)	24946.9 ± 2158.9	22922.3 ± 1585.9	29256.4 ± 3158.2
P-FFA AUC_0-30_ (mEq/ml*30min)	51.2 ± 10.5	41.0 ± 8.0	40.4 ± 7.5
HC AUC_0-30_	0.13 ± 0.01	0.15 ± 0.01	0.13 ± 0.01
*Total Phase Responses*			
Glucose AUC_0-120_ (mg/dl*120min)	19116.2 ± 734.7	18734.2 ± 760.7	18156.2 ± 780.3
Insulin AUC_0-120_ (mg/dl*120min)‡	12910 [8450, 15460]	10080 [7758, 12290]	10560 [9374, 12640]
C-peptide AUC_0-120_ (ng/ml*120min)	1368.9 ± 91.9	1313.6 ± 85.7	1349.5 ± 92.4
ISR AUC_0-120_ (ng/ml*120min)	161794.3 ± 12043.5	142435.4 ± 10755.0*	141838.1 ± 9505.4*
P-FFA AUC_0-120_ (mEq/ml*120min)	177.6 ± 41.2	136.5 ± 33.3*	120.2 ± 31.0*
HC AUC_0-120_	0.12 ± 0.01	0.13 ± 0.01	0.12 ± 0.01

Data are expressed as mean ± SEM or median (IQR) when appropriate.

‡Data log-transformed for statistical analysis. Conditions were compared by analysis of variance (ANOVA). MIE = moderate exercise intensity. HIE = high exercise intensity. FFA = free fatty acid. AUC = total area under the curve. HC = hepatic insulin clearance.

*Compared to Control, *P*<0.05.

^Compared to MIE, *P*<0.05.

### Insulin Resistance

**Fig 2:**
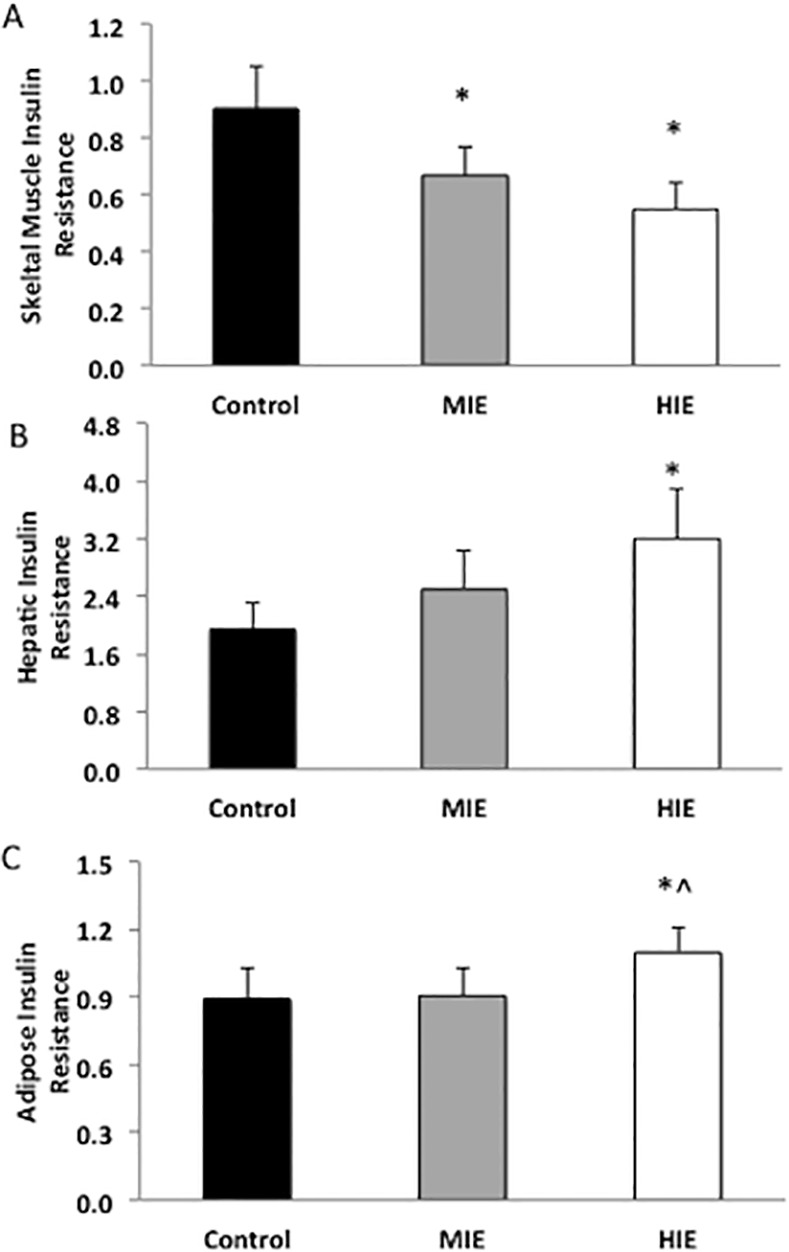
Effect of exercise intensity on multi-organ insulin resistance. Data are expressed as mean ± SEM. OMM = oral minimal model was calculated from plasma glucose and insulin to measure skeletal muscle insulin resistance. Homeostatic model of insulin resistance (HOMR-IR) was calculated as fasting PG x fasting PI to depict hepatic insulin resistance. Adipose-IR was calculated as fasting FFA x fasting PI to determine adipose insulin resistance. *Compared to Control, P<0.05. ^Compared to MIE, P<0.05.

Skeletal muscle insulin resistance decreased by 19.5±8.3% and 28.5±11.3% after moderate and high intensity exercise respectively compared with Control and independent of sex (P<0.05, Fig 2). Hepatic insulin resistance was not altered following moderate intensity exercise compared with Control, but increased following high intensity exercise (P<0.05, Fig 2). Adipose insulin resistance also rose significantly after high intensity exercise (P<0.05), but not moderate intensity exercise or Control.

### Pancreatic β-cell Function

**Fig 3:**
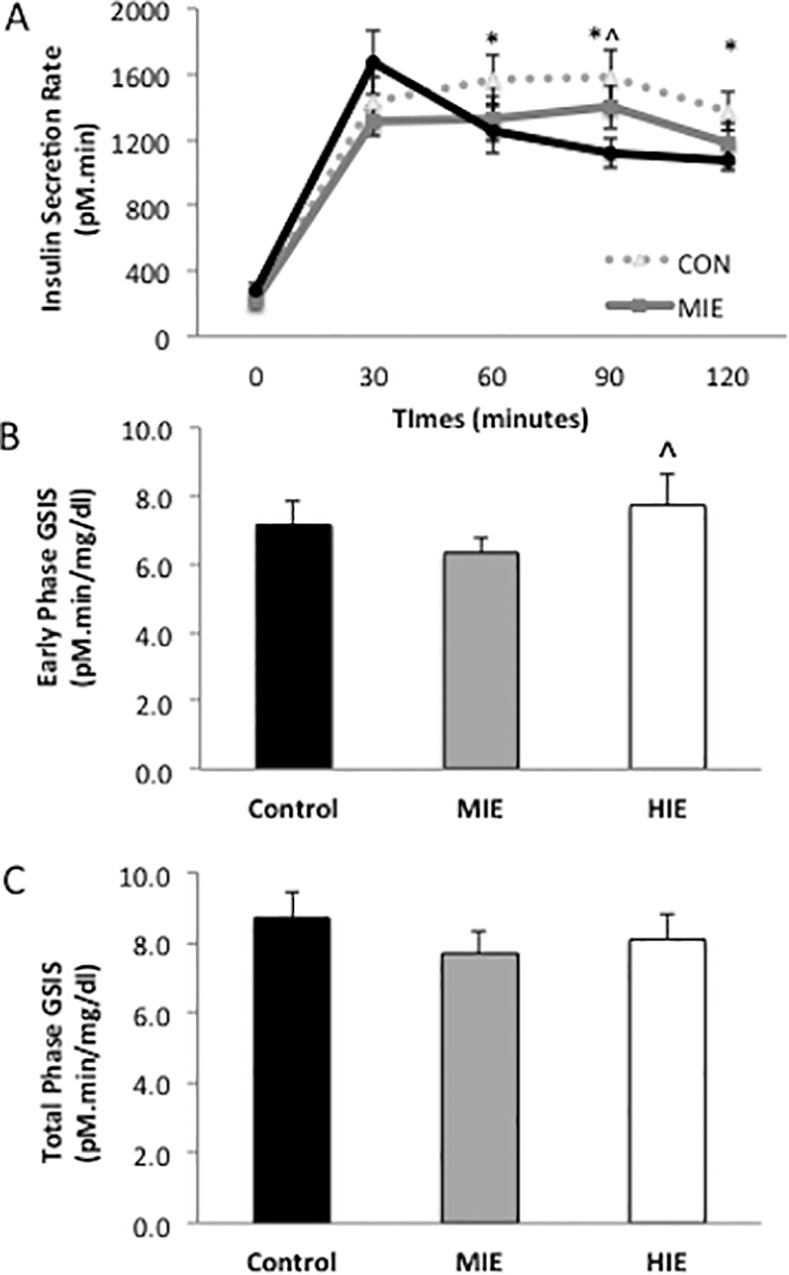
Effect of exercise intensity on glucose-stimulated insulin secretion. Data are expressed as mean ± SEM. ISR = insulin secretion rate derived from deconvolution of plasma C-peptide. GSIS = glucose-stimulated insulin secretion rate (ISR; total AUC C-peptide divided by total AUC Glucose). *Compared to Control, P<0.05.

**Fig 4:**
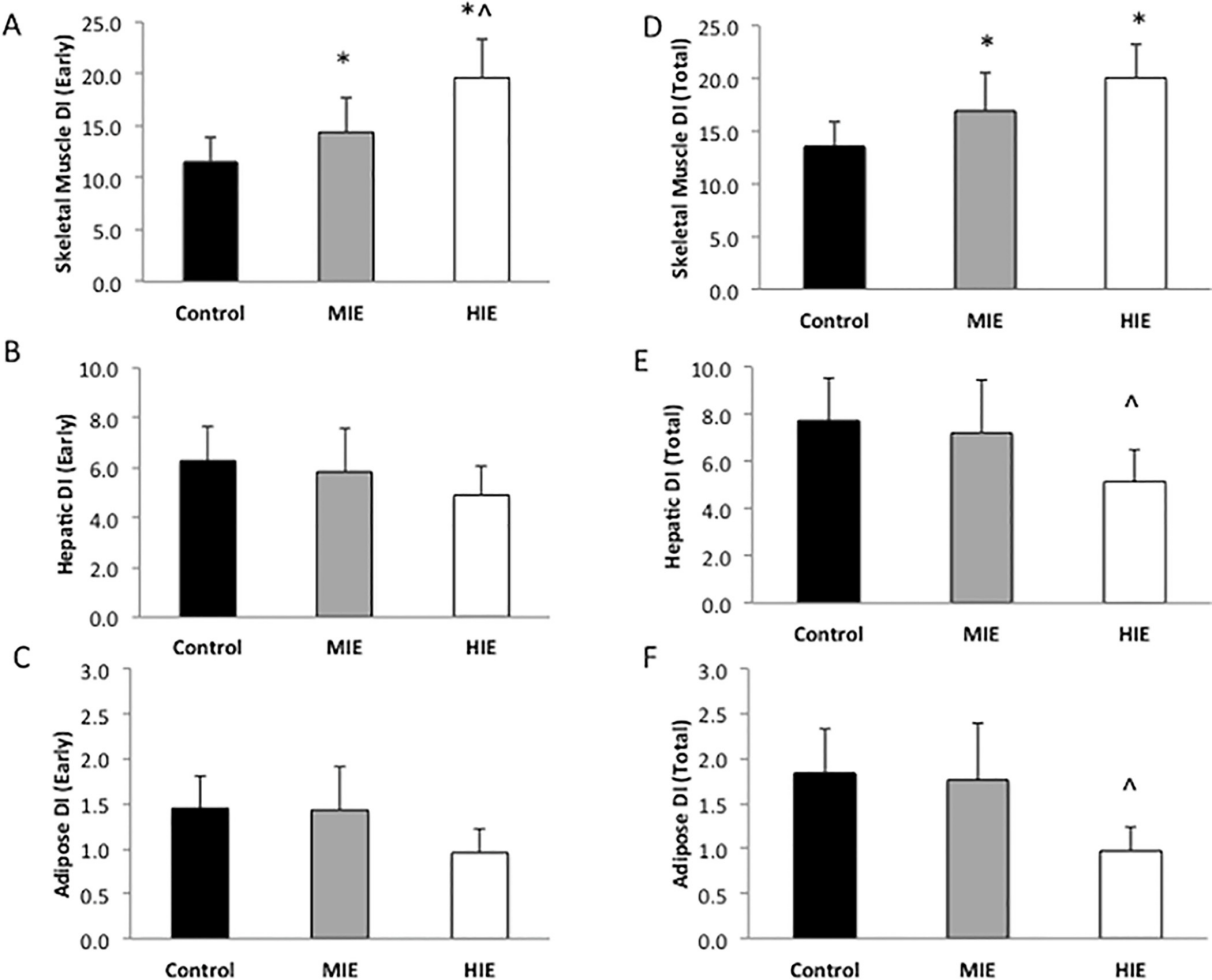
Effect of exercise intensity on β-cell function. Data are expressed as mean ± SEM. DI = disposition index and was used to characterize pancreatic β-cell function. Skeletal muscle DI was calculated as AUC of ISR/Glucose x oral glucose minimal model. Hepatic DI was estimated as AUC of ISR/Glucose x HOMA-IR. Adipose DI was determined as AUC of ISR/Glucose x Adipose-IR. *Compared to Control, *P*<0.05. ^Compared to MIE, *P*<0.05.

Early phase insulin secretion rates were increased following high intensity exercise, whereas exercise, particularly high intensity, lowered total phase insulin secretion rates compared with Control independent of sex (P<0.05, Fig 3 and Table 2). Hepatic and adipose disposition index was significantly reduced following high intensity exercise compared with Control, independent of sex (Fig 4) Moderate intensity exercise had no effect on hepatic or adipose disposition index compared with Control. Early and total phase skeletal muscle disposition index increased in an intensity based manner (Fig 4) compared with Control independent of sex.

## Discussion

The major finding from the present study is that a single high intensity exercise bout lowers GSIS relative to hepatic and adipose tissue insulin resistance when compared to an isocaloric matched moderate bout in adults with prediabetes independent of sex. In contrast, moderate and high intensity exercise increases GSIS when adjusted for changes in skeletal muscle insulin resistance compared with control. Together, these data suggest that compensatory mechanisms may exist for changes between skeletal, liver, adipose and the pancreas to support glucose homeostasis following acute exercise in an intensity based manner. Our findings are consistent with some [7,16,17] but not all [7] who report that greater volumes/intensities of exercise training increase β-cell function in overweight people. In fact, our findings confirm recent work showing that an acute bout of moderate intensity exercise has little effect on pancreatic function in people with prediabetes [18]. Prior studies reporting increases in pancreatic function following higher doses/intensities of exercise used the OGTT, whereas studies reporting little or no change used the intravenous glucose tolerance test or hyperglycemic clamp, respectively [7,16,17]. To that end, this prior work also used the intravenous glucose tolerance test, hyperglycemic and euglycemic clamp as well as HOMA to characterize insulin resistance. Notwithstanding the fact that pancreatic function derived from the clamp and intravenous glucose tolerance test are dependent on β-cell glucose responsiveness and the readily available release of insulin, whereas the OGTT also reflects the processing and synthesis of new insulin as well as evoking incretin and neural effects on the pancreas, different methodological measures of insulin resistance make comparisons between studies difficult. Our results suggest that acute exercise may independently affect GSIS in a tissue-specific manner and that the pancreatic function response after exercise may relate to the origin and/or degree of insulin resistance.

Insulin resistance promotes adjustments in insulin secretion to preserve blood glucose control [30]. In the current study, hepatic and adipose insulin resistance increased following high intensity exercise compared with control or moderate intensity exercise. While this transient insulin resistance may seem contradictory to the beneficial effects of exercise on insulin action, prior studies have reported that exercise increases hepatic glucose production and raises lipolysis in the immediate post-exercise interval in an intensity dependent manner [31–33]. In fact, elevations in growth hormone, cortisol and catecholamines provoked by high intensity exercise may contribute to this phenomenon as well as attenuate skeletal muscle glucose uptake [31–35]. However, this transient tissue-specific insulin resistance in the post-exercise period likely serves to support energy production and spare carbohydrate for glycogen restoration in skeletal muscle [31,36]. Moreover, the increase in FFA may alter hepatic lipid storage and/or insulin resistance to some extent to support blood glucose [37–39]. Nevertheless, it is important to recognize that skeletal muscle insulin resistance was significantly reduced following both moderate and high intensity exercise and blood glucose was lower in the late post-prandial period [19]. To this end, it is important to acknowledge that muscle contraction increases glucose uptake and this insulin-independent glucose disposal can persist during the post-exercise period for a few hours depending on the intensity [40]. As a result, it is likely that our observation of reduced skeletal muscle insulin resistance is to some extent exaggerated between exercise conditions by increased GLUT-4 translocation to the plasma membrane and not reflective of “true” insulin sensitivity [41,42]. Indeed, it is likely that high intensity exercise resulted in higher glycogen breakdown and promoted increased GLUT-4 compared with moderate intensity exercise thereby contributing to favorable post-prandial glucose responses. This raises important questions on the role of nutrient timing on enhancing the effect of exercise to promote insulin sensitivity. Prior studies have attempted to identify if feeding immediately post-exercise increases skeletal muscle insulin sensitivity into the next day compared with nutrient intake before or approximately 3 hours after exercise [43]. Stephens et al. [43] demonstrated that immediate feeding post-exercise had the greatest effect on skeletal muscle insulin sensitivity in overweight insulin resistant adults. Whether feeding immediately post-exercise has differential effects on insulin metabolism in an exercise intensity based manner though is unclear, particularly in a clinical population of adults with prediabetes. Herein, we show that high intensity exercise does appear to have more profound effects on insulin metabolism during the recovery period than moderate intensity exercise, and this may in part explain improved glucose tolerance.

Habitual exercise increases β-cell function in people with prediabetes [7,16] and type 2 diabetes [8,44]. In fact, prior work suggests that individuals with low baseline β-cell function are likely to improve β-cell function following exercise training [7,16]. This later point is clinically relevant, as even small amounts of exercise could benefit β-cell function [7,16]. However, whether acute exercise at a relatively low dose (i.e. 200-kcal) of different intensities modifies the insulin secretion response in people with prediabetes is not presently clear [23]. The current results show that performing a low dose of moderate or high intensity exercise may induce benefit for insulin secretion when adjusted to skeletal muscle, but not liver or adipose, insulin resistance in people with reduced β-cell function. Given the little to no change in GSIS following exercise, our results suggest that the overall net increase in insulin sensitivity may be the most important factor contributing to changes in pancreatic function following acute exercise at low doses, and skeletal muscle is likely a key driver of this process. Our current observation is consistent with prior studies assessing GSIS in conjunction with the euglycemic clamp and intravenous glucose tolerance test (which typically reflect skeletal muscle glucose disposal) and suggests that exercise may influence cross-talk between muscle and pancreas to improve glucose regulation [45–47]. However, we recognize that manipulation of nutritional composition and/or changes in physical inactivity may affect insulin secretion adjusted for liver insulin resistance [24,25].

Lipotoxicity has been implicated in the cause of pancreatic dysfunction [48–50]. The rise in FFA levels seen here with exercise may explain the lowering of hepatic and adipose disposition index estimates. Several studies show that FFA turnover is increased following high intensity exercise [31,36], and some but not all work suggests that exercise reduces circulating FFAs due to either increases in skeletal muscle intramuscular fat storage [51] and/or changes in hepatic fat accumulation [37–39]. Although the lowering of FFA concentrations in the post-exercise may contribute to whole-body improvements in pancreatic function [52], we report that FFA AUC declined similarly during the OGTT following both moderate and high intensity exercise. This suggests that FFAs per se are unlikely to have uniquely changed pancreatic function during high intensity exercise. Alternatively, recent work has suggested that elevated FFAs in the immediate post-exercise provide an energy source to peripheral organs (e.g. skeletal muscle) in effort to restore glycogen concentrations [36]. Indeed, we report significant rises in glucose AUC up to 30 minutes into the OGTT following high intensity exercise, which is consistent with increased rates of meal glucose appearance from the gut following exercise [53,54]. Interestingly enough, this rise in blood glucose is mirrored by increased insulin secretion, which explains why GSIS was not altered or slightly increased by moderate and high intensity exercise. However, the improvement in overall post-prandial glucose occurred during the later portion of the OGTT, suggesting that insulin acted in a coordinated and temporal manner to maintain glucose homeostasis. This is physiologically relevant since the disposition index was originally developed [10,29] as the inverse of GSIS and insulin resistance to reflect the integrated capacity for whole-body glucose disposal. The current work expands upon this concept and suggests that the indices used to assess insulin resistance during fasting and post-prandial conditions may offer insight to the tissues that contribute to glucose regulation [10].

There are limitations in this study that may affect our interpretations. We used surrogate measures of liver and adipose insulin resistance that may underestimate true changes in insulin action, and stable isotopes are needed to assesses skeletal muscle glucose uptake, hepatic glucose production and lipolytic rate to tease out the role of distinct tissues influencing pancreatic function. However, HOMA-IR and Adipose-IR are valid approaches to estimate hepatic and adipose insulin resistance, respectively, and surrogate measures of adipose insulin resistance and adipose disposition index correlate strongly with palmitate stable isotope turnover measures to characterize pancreatic function [55]. Moreover, the oral minimal model has been validated against the glucose clamp, which is suggested to provide insight to skeletal muscle glucose disposal whereas HOMA-IR is reflective of hepatic glucose production [12,26]. Further, glycerol may represent a more accurate biomarker of lipolysis because plasma FFA can be re-esterified and/or taken up for storage thereby limiting FFA as a lipolytic marker. It is important to acknowledge, nevertheless, that FFAs are released from adipocytes, and have a role in the development of pancreatic dysfunction [48–50]. We cannot exclude the possibility that changes in blood flow during the immediate post-exercise period may have contributed to differences in our indices of multi-organ insulin resistance. However, prior work suggests that changes in blood flow during exercise return from the periphery to the central organs (e.g. liver and adipose) within approximately 30–180 minutes post-exercise. This restoration of blood flow to adipose tissue is considered a key mechanism that accounts for elevated FFA concentrations that provide an energy source to the liver and skeletal muscle [36,56,57]. Given that FFA were not statistically different between exercise intensities 60 minutes post-exercise, differences in blood flow on indices of insulin resistance would seem minimal. Nevertheless, future work is needed following exercise to understand the role of blood flow on multi-organ insulin resistance and β-cell insulin metabolism [58]. We also used the OGTT to assess pancreatic function, and it is possible that exercise altered incretin hormones (e.g. GLP-1 and GIP) that resulted in overestimated changes in insulin secretion compared with intravenous glucose methods. Unfortunately, additives for incretin blood sample analysis were not utilized and we are unable to determine the roles of incretins on pancreatic function. However, use of oral carbohydrates increases the physiologic relevance of our study and provide “real-world” findings.

## Conclusion

Acute exercise decreases pancreatic insulin secretion relative to changes in adipose and hepatic insulin resistance in an intensity dependent manner, whereas moderate and high intensity exercise increases insulin secretion when adjusted for changes in skeletal muscle insulin resistance. Together, these data indicate that exercise intensity adjusts pancreatic insulin secretion uniquely between glucose regulatory tissues in the immediate post-exercise period to favor glycemic control in people with prediabetes. Further work is merited to understand cross-talk between skeletal muscle, liver and adipose tissue with pancreatic β-cells in order to design more effective treatments that prevent/treat type 2 diabetes.

## Supporting Information

S1 Table Data for plasma glucose, insulin, C-peptide and FFA across conditions. (PDF)

S2 Table Data for glucose-stimulated insulin secretion across conditions. (PDF)

S3 Table Data for insulin resistance across conditions. (PDF)

S4 Table Data for β-cell function across conditions. (PDF)
