# Supplementary material for: Exercise Intensity Modulates Glucose-Stimulated Insulin Secretion when Adjusted for Adipose, Liver and Skeletal Muscle Insulin Resistance
Source: PLoS One. 2016 Apr 25;11(4):e0154063. doi: 10.1371/journal.pone.0154063 (PMC4844153; doi:10.1371/journal.pone.0154063)
Supplement: S1 Table — (PDF) [file pone.0154063.s001.pdf]

S1 Tables:

Glucose Concentrations (mg/dl)

	TIME	0	30	60	90	120
Control	mean	92.1	145.4	176.2	182.7	173.7
MIE	mean	92.6	152.2	174.3	170.9	161.7
HIE	mean	95.6	163.2	164.7	155.3	148.6
Control	SEM	1.8	5.8	7.3	11.0	9.1
MIE	SEM	2.7	5.7	8.1	10.1	9.8
HIE	SEM	3.0	7.0	10.6	7.7	8.7

Insulin Concentrations (uU/ml)

	TIME	0	30	60	90	120
Control	mean	8.4	94.1	127.0	153.6	153.0
MIE	mean	10.5	79.9	113.7	130.3	140.6
HIE	mean	12.8	115.4	123.8	117.0	107.2
Control	SEM	1.6	12.3	17.7	24.1	24.8
MIE	SEM	2.1	9.9	19.5	23.6	29.4
HIE	SEM	2.5	17.4	21.9	22.8	16.9

C-peptides (ng/ml)

	TIME	0	30	60	90	120
Control	mean	2.5	8.7	12.6	15.2	15.8
MIE	mean	2.9	9.1	12.3	13.9	14.2
HIE	mean	3.4	10.7	13.0	13.1	13.1
Control	SEM	0.2	0.7	0.9	1.2	1.2
MIE	SEM	0.3	0.6	0.9	1.1	1.2
HIE	SEM	0.3	0.8	1.2	1.0	1.0

FFA (mEq/ml)

	TIME	0	30	120
Control	mean	1.5	1.9	0.9
MIE	mean	1.2	1.5	0.6
HIE	mean	1.5	1.2	0.6
Control	SEM	0.3	0.4	0.3
MIE	SEM	0.2	0.3	0.2
HIE	SEM	0.2	0.3	0.2
