# Supplementary material for: Exercise Intensity Modulates Glucose-Stimulated Insulin Secretion when Adjusted for Adipose, Liver and Skeletal Muscle Insulin Resistance
Source: PLoS One. 2016 Apr 25;11(4):e0154063. doi: 10.1371/journal.pone.0154063 (PMC4844153; doi:10.1371/journal.pone.0154063)
Supplement: S2 Table — (PDF) [file pone.0154063.s002.pdf]

S2 Tables:

Skeletal Muscle Insulin Resistance

	Mean	SEM
Control	0.9	0.15
MIE	0.7	0.09
HIE	0.5	0.09

Hepatic Insulin Resistance

	Mean	SEM
Control	1.9	0.3
MIE	2.5	0.5
HIE	3.2	0.6

Adipose Insulin Resistance

	Mean	SEM
Control	0.89	0.14
MIE	0.90	0.13
HIE	1.10	0.11
