# Supplementary material for: Exercise Intensity Modulates Glucose-Stimulated Insulin Secretion when Adjusted for Adipose, Liver and Skeletal Muscle Insulin Resistance
Source: PLoS One. 2016 Apr 25;11(4):e0154063. doi: 10.1371/journal.pone.0154063 (PMC4844153; doi:10.1371/journal.pone.0154063)
Supplement: S3 Table — (PDF) [file pone.0154063.s003.pdf]

S3 Tables:

Insulin Secretion Rate

	TIME (min)	0	30	60	90	120
Control	mean	226.7	1436.5	1566.9	1590.2	1372.5
MIE	mean	218.7	1309.5	1331.4	1405.2	1184.8
HIE	mean	275.5	1675.0	1258.6	1115.8	1081.6
Control	SEM	26.7	142.9	147.4	165.1	114.0
MIE	SEM	31.0	92.8	138.1	135.9	117.3
HIE	SEM	49.8	198.8	145.3	88.8	72.3

GSIS early phase

	Mean	SEM
Control	7.2	0.7
MIE	6.3	0.5
HIE	7.7	0.9

GSIS total phase

	Mean	SEM
Control	8.7	0.7
MIE	7.7	0.5
HIE	8.1	0.7
