# Supplementary material for: Exercise Intensity Modulates Glucose-Stimulated Insulin Secretion when Adjusted for Adipose, Liver and Skeletal Muscle Insulin Resistance
Source: PLoS One. 2016 Apr 25;11(4):e0154063. doi: 10.1371/journal.pone.0154063 (PMC4844153; doi:10.1371/journal.pone.0154063)
Supplement: S4 Table — (PDF) [file pone.0154063.s004.pdf]

*S4 Tables:*

**Skeletal Muscle Disposition Index early phase**

| | Mean | SEM |
|---|---|---|
| Control | 1.5 | 0.3 |
| MIE | 1.4 | 0.4 |
| HIE | 1.0 | 0.2 |

**Hepatic Disposition Index early phase**

| | Mean | SEM |
|---|---|---|
| Control | 6.3 | 1.4 |
| MIE | 5.8 | 1.7 |
| HIE | 4.9 | 1.2 |

**Adipose Disposition Index early phase**

| | Mean | SEM |
|---|---|---|
| Control | 1.5 | 0.3 |
| MIE | 1.4 | 0.4 |
| HIE | 1.0 | 0.2 |

**Skeletal Muscle Disposition Index Total**

| | Mean | SEM |
|---|---|---|
| Control | 13.5 | 2.3 |
| MIE | 16.9 | 3.5 |
| HIE | 20.0 | 3.2 |

**Hepatic Disposition Index Total**

| | Mean | SEM |
|---|---|---|
| Control | 7.7 | 1.7 |
| MIE | 7.2 | 2.2 |
| HIE | 5.1 | 1.3 |

**Adipose Disposition Index Total**

| | Mean | SEM |
|---|---|---|
| Control | 1.8 | 0.4 |
| MIE | 1.8 | 0.6 |
| HIE | 1.0 | 0.2 |
